# Volatile Compounds Content, Physicochemical Parameters, and Antioxidant Activity of Beers with Addition of Mango Fruit (*Mangifera Indica*)

**DOI:** 10.3390/molecules25133033

**Published:** 2020-07-02

**Authors:** Alan Gasiński, Joanna Kawa-Rygielska, Antoni Szumny, Anna Czubaszek, Justyna Gąsior, Witold Pietrzak

**Affiliations:** 1 Department of Fermentation and Cereals Technology, Faculty of Biotechnology and Food Science, Wrocław University of Environmental and Life Science, Chełmońskiego 37 Street, 51-630 Wroclaw, Poland; joanna.kawa-rygielska@upwr.edu.pl (J.K.-R.); anna.czubaszek@upwr.edu.pl (A.C.); justyna.gasior@upwr.edu.pl (J.G.); witold.pietrzak@upwr.edu.pl (W.P.); 2 Department of Chemistry, Faculty of Biotechnology and Food Science, Wrocław University of Environmental and Life Sciences, C. K. Norwida street 25, 50-375 Wrocław, Poland; antoni.szumny@upwr.edu.pl

**Keywords:** *Mangifera indica*, beer, solid-phase microextraction, in-needle SPME, total polyphenols conent, volatile compounds, gas-chromatography

## Abstract

This study was performed to determine the possibility of using mango fruit (*Mangifera indica*) in brewing technology. The aim of using the SPME-HS-GC-MS technique was to assess what changes occurred in the volatile composition of mango beers brewed in this study. Mango fruit was added to the beer in five different forms to ascertain what kind of preparation should be used to improve beer aroma. Analysis of the volatile components in mango beer showed that beer without mango addition was characterized by the lowest content of volatile compounds (1787.84 µg/100 mL). The addition of mango fruit increased the concentration of compounds, such as α-pinene, β-myrcene, terpinolene, α-terpineol, *cis*-β-ocimene, caryophyllene, and humulene, in beer. Beer prepared with mango pulp addition was characterized by the highest concentration of volatile components from mango beers (2112.15 µg/100 mL). Furthermore, beers with mango addition were characterized by a higher polyphenol content (up to 44% higher than control beer) and antioxidant activity than control beer and were evaluated by a trained panel as having a better taste and aroma than beer without fruit addition.

## 1. Introduction

Beer is one of the oldest alcoholic beverages in the world and it is produced all over the world. It is produced through alcoholic fermentation of beer wort, made mostly from barley malt, carried out by yeast. Beer is rich in many valuable human diet substances, such as amino acids, vitamins, minerals, and phenolic compounds [1,2]. Most of these compounds come from malt, but hops add a small (20–30% of all phenolic compounds in beer) but significant portion of polyphenols [3]. Phenols and polyphenols can contribute to such characteristics of beer as the flavor, haze, fullness, and astringency [4,5]. Volatile components in beer have a far greater impact on the flavor characteristics than phenolic compounds. Beer is a beverage with a complex content of volatile components, which belong to various chemical classes, such as alcohols, volatile phenols, esters, fatty acids, terpenoids, and C13-norisoprenoids [6]. There are three main contributors to the volatile compounds’ concentration in beer. They are, as in the case of phenols, malt and hops, but many of the flavor active volatiles in beer are by-products of yeast metabolism [7,8]. The global beer market is mostly dominated by traditional types of beer, but an increase in interest in beers made with the addition of fruit can be noticed. In addition, the consumption of exotic fruit is rising worldwide, probably due to increasing public awareness of their nutritional and health properties [9]. In the last years, few studies of beers with fruit addition have been carried out and it has been shown that the addition of fruit to beer is possible and it can improve the health properties of manufactured beer; however, these studies have not emphasized changes in the volatile composition of beers produced with fruit addition [10]. Moreover, in those studies, authors have not used one of the most popular fruits consumed in the world, mango (*Mangifera indica*). Mango has been cultivated for over four millennia and is second only to pineapple in value and quantity among tropical fruits [11]. It is a fruit rich in many various chemical components. It is a source of phenolics, such as quercitin derivatives, flavonoids, gallotannins, ellagic acid derivatives, gallates, xanthones (mainly mangiferin), and benzophones. Other phytochemicals in mango are carotenoids and anthocyanins. Mango is also a source of many vitamins, such as ascorbic acid, niacin, or folic acid. Moreover, mango fruits contain many volatile compounds (various esters, monoterpenes, sesquiterpenes, and lactones), which are characterized by a sweet fruity aroma [12]. The combination of these characteristics shows that mango fruit could be used as an adjunct to beer, which would not only create a more complex and rich aroma but also improve the health benefits of manufactured beer. The goal of this study was to determine what form of mango fruit addition would be best suited to improve the flavor of produced beer.

## 2. Results and Discussion

### 2.1. Concentration of Volatile Compounds in Tested Beers

In this study, beers with the addition of mango fruit (*Mangifera indica*) in different versions were tested to assess what type of addition would have the greatest impact on the composition of volatile compounds in the final products. Five types of beer with mango and control beer without fruit were prepared. To each fruit beer, mango was added in the same amount and at the same time but in a different form (juice, pulp, raw/heatedhomogenisate, raw pieces). In the tested beers, 68 volatile compounds were identified (Table 1). They were divided into 11 main chemical groups. The largest of them were esters (28 compounds), followed by alcohols (12 compounds) and monoterpenes (10 compounds). Hydrocarbons (5 compounds), acetals (3 compounds), phenylpropanoids (3 compounds), sesquiterpenes (2 compounds), aldehydes (2 compounds), acids (one compound), and ketones (one compound) were also identified. Analysis revealed one peak, which was identified as a volatile constituent, but it could not be identified (the mass spectra of the unidentified constituents and total ion chromatogram of MP are available in Figures S1 and S2). The content of five compounds identified in mango fruit, such as α-pinene, camphene, p-cymene, terpinolene, and humulene, was determined in all tested beers, although the concentration of them was, with the exception of humulene, far smaller in beer without the mango addition (BC). It is worth noting that these compounds do not belong to the largest chemical groups of volatile compounds in the tested beers. The largest concentration of compounds characteristic for mango fruit and the largest amount of volatile compounds in total was recorded for beer with mango pulp addition (MP).

The compound derived from mango fruit, which was found in the largest amount in MP, was terpinolene, characterized by a sweet citrusy aroma. It is a constituent of many essential oils and can be used as a flavoring agent [13]. It is a compound, which is commonly found in mango fruits, especially in cultivars grown on the American continent [14]. Its concentration was demonstrated in all tested samples, but in MP (73.162 µg/100 mL), it was far greater than in BC (0.880 µg/100 mL). Another volatile monoterpene, found in mango fruit, was camphene. As in the case of terpinolene, the largest concentration was found in MP (0.593 µg/100 mL) and the smallest in BC (0.013 µg/100 mL). Camphene is characterized by a piney, woody, and citrusy aroma [15]. P-cymene, which is also characterized by a citrusy and woody aroma, but is not a monoterpene as camphene, was found in the greatest concentration in MP (2.449 µg/100 mL).

Another compound identified in mango fruit was α-pinene, which is a monoterpene with a characteristic piney turpentine-like aroma [16]. The greatest concentration of α-pinene was recorded for MP (5.764 µg/100 mL) and the smallest for BC (0.660 µg/100 mL). It is worth noting that α-pinene and another compound, humulene, are found not only from mango fruit but are important components of hop (*Humulus lupulus*) cones, which are used to create a characteristic beer aroma [17]. Humulene is a sesquiterpene produced by many aromatic plants, such as pine trees, tobacco, hemp, and hops, but it can also be found in the flesh and skin of mango fruit. In the tested beers, the highest concentration of humulene was found in MP (33.441 µg/100 mL) and the smallest in BC (25.230 µg/100 mL). Humulene is characterized by a woody aroma [18]. Another compound, typically found in hop cones and detected in all tested beers, was β-myrcene. Humulene is a compound found in many aromatic plants, such as pine, salvia, ginger, and cumin. It is a component of many essential oils [19]. It was found in all tested beers, in the tgreatest amount in MP (68.384 µg/100 mL) and beer with mango juice (MJ) (67.152 µg/100 mL). The smallest amount of β-myrcene was recorded in BC (51.683 µg/100 mL). It is worth noting that, despite the lowest content of volatile compounds in total, the concentration of several components is higher in the BC than in any of the tested beers with the addition of mango fruit. The highest concentration of 3-methyl-1-butanol was recorded in BC (223.169 µg/100 mL), while the lowest was noted for MJ (177.716 µg/100 mL). It is one of the higher alcohols (also known as fusel alcohols) produced by yeast, which can, depending on the concentration, have both a positive and negative impact on the flavor and aroma of beer [20]. Another compound, the concentration of which was highest in BC (216.684 µg/100 mL), was phenylethyl alcohol. It is a substance that can be produced by yeast from amino acid L-phenylalanine. It is characterized by a flowery rosy aroma [21]. The lowest concentration was found in MJ (184.237 µg/100 mL). The total concentration of all volatile compounds in the tested beers was highest for MP (2112.147 µg/100 mL) and the lowest for BC (1787.836 µg/100 mL). It is worth noting that an increased concentration of volatile components does not always result in a better beer aroma. The aroma of many chemical substances, such as diacetyl, can be perceived as a defect in beer or some of beer styles. There are also volatile components characterized typically by pleasant aromas, which at a concentration that is too high start to lose their well-perceived fragrances and worsen the beer’s acceptability. This is why it is crucial not to only examine the total amount of volatile compounds in beers but to assess how changes in beer brewing technology, such as the addition of fruit, modify the concentration of individual chemicals [22]. Differences that can be seen between the different additions of mango to the beer on the concentration of volatile components can be explained by the way in which mango is processed. MR, MRH, and MHH were characterized by a lower concentration of compounds identified in mango fruit. They were prepared by adding fresh mango preparations. Mango is a climacteric fruit, which means that it can ripen after it is harvested from the tree, in shipping and in storage. It allows for the transportation of fruit overseas, but mango that is harvested after it has achieved its maturity on the tree is characterized by a higher content of volatile components [23]. Mango pulp and mango juice are prepared from freshly harvested fruit and this might be a reason why MF and MJ are characterized by a greater content of volatile components, which come from mango. MJ has a lower content of volatile compounds than MF, because mango juice is essentially mango pulp that has gone through a more effective filtration/pressing process, which could remove particles rich in volatile compounds [24]. Among the beers with the addition of fresh mango (MR, MHH, MRH), MR was characterized by the lowest concentration of volatile compounds. This is probably due to the fact that homogenisation breaks the walls of some mango cells, which releases their content into the solution. Another factor that could explain the higher concentration of volatile compounds in MRH and MHH than in MR is the increased surface contact between particles of fruit and beer, which improves the transfer of acids, phenols, sugars, alcohols, terpenes, and vitamins into the beer. MHH was characterized by a lower content of volatile compounds than MPH probably due to the evaporation of some volatiles during the heating process [25].

### 2.2. Concentration of Carbohydrates and Glycerol

The method used in this study to examine the carbohydrate profile and glycerol concentration in the tested beers was HPLC, by which the contents of dextrins, maltotriose, glucose, and maltose were assessed (Table 2).

The carbohydrate content in the tested beers ranged from 27.72 to 34.91 g/L. The highest concentration among the analyzed carbohydrates was found for dextrins. Dextrins in beer come from malt and are carbohydrates that cannot be utilized by the brewing yeast used in this study [26]. The concentration of dextrins in ripe mango fruit is lower than in beer wort; therefore, the addition of mango to beer should decrease the dextrin concentration, as it was shown in the conducted study [12]. The use of mango pulp, raw mango and mango homogenisates did not decrease the content of sugars as much as the use of mango juice, because after the fermentation process, the fruit solids were separated from the beer. Because of this, the use of mango juice diluted the volume of the beer compared to other mango additions. The glucose content was not detected in any of the beers. It is a sugar, which is preferentially used by *Saccharomyces cerevisiae* yeast. Beer usually does not contain glucose or contains only a minuscule amount of it [27]. The maltose content was only noted for BC and MR and in each of these beers it was similar. Similarly, maltotriose was also found in BC and MR, but its content was three times higher in BC. The composition and content of carbohydrates is an important factor, which may have an impact on the organoleptic characteristics of beer. It is also a critical element forming the properties of fruit and fruit preparations by having an effect on the final taste of the product and thus on the reception of the product by consumers [28]. The glycerol content in the analyzed beers ranged from 1.95 to 2.31 g/L. Glycerol is a chemical compound, which has an influence on the sensory traits of fermented beverages. It affects such characteristics as the viscosity of beer, its palatability, and the sensation of sweetness [29].

### 2.3. Physico-Chemical Properties of Beer

The basic physico-chemical properties of beers with mango addition were tested by densimetry, near-infrared spectroscopy, and potentiometry (Table 3).

All beers with the addition of mango were characterized by a lower pH value than BC. Mango fruits are rich in many organic acids and their pH is lower than the pH of traditional beer. The main organic acids, which are necessary for the proper metabolism of fruit, are malic acid and citric acid [30]. The content of acids in mango fruit changes throughout the ripening process, so there is a possibility that fruit used for MR, MRH, and MHH, which had the lowest pH value from tested beers, were not as ripe as fruit used to produce mango pulp and juice used for MP and MJ.

The pH of mango beers in this study was similar to the pH of other fruit beers, like beers with cornelian cherry juice analyzed by Kawa-Rygielska et al. [31], which achieved a pH level in the range of 3.43–7.71, or fruit beers analyzed by Nardini and Garaguso [10], which also achieved a pH as low as 3.5. PH of the beer. This is related to its microbial stability, because at lower pH, the probability of growth of unwanted microorganisms decreases, so it can be seen as an advantage by a manufacturer. Nevertheless, modification of the technological process to add fruits at a later stage of beer fermentation may increase the risk of beer contamination, so due caution over this additional step in beer manufacture is needed [32]. Analysis of the physico-chemical properties showed that BC and MJ achieved the lowest alcohol content. Beers with mango solids added to them were characterized by a higher alcohol content than BC. It is worth noting that MJ, despite achieving a similar alcohol content to BC, was characterized by a higher degree of fermentation. The main reason for this was probably the composition of fruit juice, which consists of less non-fermentable sugars than typical beer wort, so despite diluting the beer, it also decreased the content of carbohydrates that could not be used by yeast. The degree of fermentation is significant in beer brewing technology, because it determines the efficiency of alcohol production; therefore, proper control of that factor is of utmost importance. A higher alcohol content is typical for fruit beers, as was proved in many publications about this type of alcoholic drink.

Most of the mango beers (MJ, MP, MRH, MHH), despite a similar or higher alcohol content than BC, contained less calories due to the higher degree of fermentation and lower sugar concentration. The real extract of all mango beers was also lower than the extract of BC, which is another factor that can improve beer microbial stability.

### 2.4. Concentration of Polyphenols and Antioxidative Activity

In this study, the Folin–Ciocalteu method, DPPH**^•^**, ABTS**^+•^**, and FRAP assay were used to determine the tpolyphenol concentration and antioxidant activity in the tested beers (Table 4).

Among the analyzed beers, the highest total polyphenol content was noted for MJ—it was higher by 44% than in BC. The DPPH**^•^**, ABTS**^+•^,** and FRAP tests also showed that MJ had the highest antioxidant activity from all tested beers. The results of this study showed that the addition of mango fruit in all used forms increased the polyphenol concentration in prepared beer. It also showed that homogenising fruit prior to addition to beer will result in a greater increase of the polyphenols and antioxidant activity than adding fruit in bigger pieces. This is probably caused by the higher area of contact with the surrounding beer and the breakage of plant cell walls, which can help in releasing phenolic compounds into the solution. The preparation and heat treatment of mango juices was also assessed by Dars et al. [33] and is consistent with the results acquired in our study. Juices acquired from mango, which were acquired from well-homogenized fruit, were characterized by a higher polyphenol content. The heat treatment of homogenized mango reduced the concentration of phenolic compounds. These factors may explain why MRH has higher antioxidant activity than MR and MHH. The higher polyphenol content in fruit beers than in traditional beers achieved in this study is consistent with previous research about beer with goji berries conducted by Ducruet et al. [34] or with beer with cornelian cherry conducted by Adamenko et al. [35], although mango fruit caused a smaller increase of the polyphenols or antioxidant activity than in the mentioned research. The reason for this might be the smaller content of polyphenols in mango fruit than in goji berries in cornelian cherry, which are fruits characterized by exceptionally high contents of natural antioxidants. In comparison, in a study about beer with persimmon fruit, which does not have a high polyphenol content, conducted by Martinez et al. [36], beer with fruit addition was characterized by a lower content of polyphenols than the beer with mango addition analyzed in this study.

### 2.5. Organoleptic Analysis

The best rated beer in the criteria of aroma, color, taste, and overall impression was MP (Table 5). The only criterion in which MP achieved worse notes than BC was beer clarity. All beers with fruit addition were characterized by a better aroma. Only MR achieved the same lowest note in the criterion of beer color as BC.

Testers were also asked to describe the aromas they sensed in the tested beers. BC was described as having a bread-like, straw-like, and grainy aroma. MJ was described as having a sweet, fruity, and pineapple-like aroma. MP was described as having an intensively fruity aroma. Three of the testers described MP as a beer having a very rich, sweet, and mango-like aroma. The MR aroma was described as woody, piney, and flowery with bread-like tones. MRH was described as having a sweet fruity aroma with hints of pine sap and rose flowers. MHH was described mostly as having a sweet, fruity, and pine aroma.

Studies about the sensory properties of beer with fruit addition conducted in the past years by Adadi et al. [32] also demonstrated that significant changes in the character of beer can be noted the after addition of fruit and consumers mostly choose fruit beers as being more desirable compared to traditional types of beer.

In the study conducted by the Viejo et al., which used neural networks and advanced machine learning connected with state-of-the-art chromatographic analysis, showed that beers that were characterized by a greater concentration of volatiles with fruity, floral, and sweet aromas were preferred by the consumers because of their better taste and more pleasant aroma [37].

## 3. Materials and Research Methods

### 3.1. Materials

#### 3.1.1. Reagents and Standards

Reagents used in this study were diammonium salt of 2,2-azobis (3-ethylbenzothiazoline-6-sulfonate) (ABTS^+^**^•^**), 1,1-diphenyl-2-picrylhydrazil (DPPH**^•^**) radical, 2,4,6-tripyridyl-S-thiazine (TPTZ), 20% aqueous sodium carbonate solution, Folin-Ciocalteu reagent, acetic acid, sulfuric acid, FeCl_3_, sodium acetate, and diatomaceous earth. The internal standard used for gas chromatography was 2-undecanone (Sigma-Aldrich, Saint Louis, MO, USA) with a content of 100 mg of compound per 100 mL of distilled water.

#### 3.1.2. Biological Material

*Saccharomyces cerevisiae* US-05 from Fermentis company (Lesaffre, France) was used to ferment wort. Yeast was rehydrated in sterile water prior to inoculation for 20 min. It was added into wort in the amount recommended by the producer (0.58 g d.m./1 L of wort).

#### 3.1.3. Raw Material

The raw fruit material used in this study was mango fruit of cultivar Palmer harvested in Brazil, mango juice from company Ekamedica (Kozy, Poland), and mango pulp from a company Panagera (Miraflores, Portugal). The raw material used to brew beer was Pilzen malt from Viking Malt company (Strzegom, Poland) and Magnum hop pellets with an alpha acid content of 11.5% (*w*/*w* dry mass).

#### 3.1.4. Research Material

The research material was beer in six variants: Without any additives (BC), with the addition of mango juice (MJ), with the addition of mango pulp (MP), with the addition of cubes of raw mango (MR), with the addition of raw mango homogenisate (MRH), and with the addition of heated mango homogenisate (MHH).

### 3.2. Brewing Technology

Mashing was carried out under laboratory conditions with an infusion system in the following conditions: 67 °C for 70 min. Next, the whole mash was heated to 78 °C to inactivate the malt enzymes, filtered, and 21 L of wort were obtained. The wort was boiled for 60 min with the addition of hops at the start of the boiling. The hopped wort was cooled to 25 °C, filtered, and aerated. The initial extract content was set at 11.5°Plato, measured using a Densito 30PX densimeter (Mettler Toledo, DC, USA). Fermentation was carried out in one fermentation vessel for the first seven days. After this period, beer was transferred to separate 1-L glass fermentation flasks, to which fruit addition (with exception of BC) was added. Into every version of mango beer, 20% (*w*/*w*) of mango was added. In the case of MR, mango was peeled, and its flesh was cut into cubes with each edge 1 cm long. Mango added to MRH was peeled and its flesh was homogenized using an electric blender. A similar procedure was used for MHH, but mango flesh, after homogenisation, was added to a glass beaker and heated to 70 °C in a water bath and held at this temperature for 10 min. Fermentation was continued after fruit addition for another 10 days, then beer was filtered through filter paper MN 614 ¼ from company Macherey-Nagel (Düren, Germany) and bottled. Fermentation was carried out at a temperature of 22 °C.

### 3.3. Analytical Methods

#### 3.3.1. Adsorption of Volatile Compounds Using Solid-Phase Microextraction (SPME)

Adsorption of volatile compounds was carried out according to a modified version of the method used by Łyczko et al. [38]. Centrifuged (6000 rpm, 10 min-2675 centrifugal force) and degassed beer (2 mL) was added to a 20-mL glass vial. Then, 5 µL of 100 mg/100 mL aqueous emulsion internal standard solution (2-undecanone) were added. A magnetic stir bar was placed in the vial, which was then sealed with an aluminum membrane. The vial was placed on the heatplate of an IKA RCT Basic (Staufen, Germany) magnetic stirrer. The SPME holder needle with three-component universal fiber for SPME (DVB/CAR/PDMS 50/30 µm) was used to pierce through the membrane. Heating to 40° C and stirring was started and the fiber was extended and held over the beer for 30 min. An analogical approach was used for each type of beer. The adsorption of volatile compounds contained in mango fruit flesh, mango fruit juice, and mango fruit pulp added to beer was performed in order to find out which chemical compounds are characteristic for mango fruit. The adsorption of compounds isolated from the mango fruit was carried out in an analogous manner to the adsorption of volatile compounds from tested beers, but 2 g of mango pulp or fruit flesh were used instead of 2 mL of beer. In case of mango juice, 2 mL were used instead of 2 mL of beer.

#### 3.3.2. Gas Chromatography and Mass Spectrometry of Compounds Adsorbed on SPME Fiber

Separation, quantification, and identification of volatile compounds adsorbed on the fiber was carried out using a gas chromatograph connected to a Saturn 2000 MS Varian Chrompack mass detector (Palo Alto, CA, USA) with a ZB-5 column (Phenomenex, Torrance, CA, USA) (30 m length × 0.25 µm film thickness × 0.25 mm diameter). Chromatographic conditions were carried out in accordance with the methodology of Calin-Sanchez et al. [39]. Scanning (1 scan/s) was carried out in the 35–400 *m*/*z* range using 70 mV electron ionization. The analyses were carried out with the use of helium as a carrier gas with a flow rate of 1 mL/min using the following program for the oven temperature: 40 °C at the beginning of the process, 5 °C/min up to 110 °C, and 20 °C/min up to 270 °C. The initial temperature was maintained for 3 min. The injection port temperature of the chromatograph was 220 °C.

#### 3.3.3. Carbohydrate Profile and Glycerol Content

The sugar profile and the content of glycerol were examined by means of high-performance liquid chromatography (HPLC) [40]. Beer samples were degassed and centrifuged (6000 rpm, 10 min) and then were diluted (1:1) with ultrapure water and filtered through syringe nylon filters (0.45 µm pore size) to chromatographic vials. The samples were then analyzed using a Prominence liquid chromatography system (Shimadzu Corp., Kyoto, Japan) equipped with a Rezed ROA-Organic Acid H + column (300 × 4.6 mm) from Phenomex (Torrance, CA, USA). The following measurement parameters were used: Sample volume: 20 µL; separation temperature: 60 °C; mobile phase flow rate: 0.6 mL/min; mobile phase: 0.005 M H_2_SO_4_; and detection temperature: 50 °C. The concentration of ethanol, glycerol, dextrins, maltose, glucose, and maltotriose was based on five-point calibration curves using Chromax 10.0 software (Pol-Lab, Wilkowice, Poland). Ten measurements were performed for each type of tested beer.

#### 3.3.4. Basic Physico-Chemical Parameters

The concentration of ethyl alcohol, extract content, density, wort extract, and calorie content in mango beers was analyzed using an Anton Paar DMA 4500M oscillating densitometer (Graz, Austria). Density of samples was measured using oscillating U-tube, while the alcohol content was analyzed by near infrared (NIR) spectroscopy. Prior to analyses, the samples of beer were degassed and centrifuged (6000 rpm, 10 min) and then filtered through laboratory filer paper.

The pH value of the beer was measured with an MP 240 Mettler Toledo pH meter (Columbus, OH, USA). The calorie value of the beer was calculated on the basis of the density (ρ), real extract content (Er), and alcohol content (A), according to the equation below. All analyses were carried out in three replications. Calorie content [kcal/100 mL] = (7 × A_(%*w*/*w*)_ + 4 × Er_(%*w*/*w*)_ × ρ).

### 3.4. Analysis of Total Polyphenols Content and Antioxidative Activity

#### 3.4.1. Analysis of Total Polyphenols Content

The total polyphenol content of the beers in this study was analyzed using the Folin-Ciocalteu (F-C) spectrophotometric method [41]. First, 0.1 mL of degassed and centrifuged beer sample and 0.2 mL of F-C reagent were pipetted into cuvettes. After 3 min, 1 mL of a 20% aqueous solution of sodium carbonate (Na_2_CO_3_) and 2 mL of distilled water were added. The absorbance was measured at the 765-nm wavelength after 1 h of incubation at room temperature using a Beckmann DU650 spectrophotometer (Brea, CA, USA) and the results were expressed as mg of gallic acid equivalents (GAE) per L of beer. Measurements were performed in 12 replications.

#### 3.4.2. Free-Radical-Scavenging Ability by the Use of a DPPH^•^ Radical

The first method to measure the antiradical activity of beers prepared in this study was a DPPH^•^ radical assay [42]. First, 0.1 mL of beer sample was pipetted into polystyrene cuvettes and mixed with 2 mL of 0.04 mmol/L DPPH^•^ dissolved in ethanol and 0.4 mL of distilled water. After 10 min of incubation at room temperature, the absorbance was measured with a spectrophotometer at 517 nm. A calibration curve was prepared with Trolox solution (0.005 mmol/L). The data were expressed as Trolox equivalent (TE) of antioxidative capacity per 1 L of the beer (mmol TE/L). All measurements were performed in 12 replications.

#### 3.4.3. Ferric Reducing/Antioxidant Power (FRAP) Assay

The FRAP assay is based on the reduction of ferric 2,4,6-*tris*(2-pyridyl)-1,3,5-triazine [Fe (III)-TPTZ] to the ferrous complex at low pH, which is analyzed by a change of absorbance measured by a spectrophotometer [43]. The reagent was prepared by mixing 10 mmol 2,4,6-*tris*(2-pyridyl)-s-triazine (TPTZ)/L reagent with 20 mmol/L ferric chloride in acetate buffer (acetic acid and sodium acetate solution with pH 3.6). Quantitative analyses were performed by the external standard method using ferrous sulphate (0.2 mmol/L) as the reference standard and correlating the absorbance (wavelength 593 nm) with the concentration. Then, 0.1 mL of beer sample was mixed in polystyrene cuvettes with 0.9 mL of distilled water and 3 mL of ferric complex. The results were calculated and expressed as milimoles of Trolox per 1L of beer. All measurements were performed in 12 replications.

#### 3.4.4. Free-Radical-Scavenging Ability by the Use of an ABTS^+^**^•^** Radical Cation

Another method used to measure the antioxidant activity of beers was the ABTS^+^**^•^** radical cation assay [44]. First, 0.03 mL of beer sample was mixed with 3 mL of ABTS^+^**^•^** solution with measured absorption of 0.700 at a wavelength of 734nm. After 6 min of incubation, the absorbance of the samples was measured. Each sample was tested in 12 replications. The data were expressed as mmol Trolox equivalent of antioxidative capacity per 1 L of the beer (mmol TE/L).

### 3.5. Sensory Analysis

The mango beers prepared in this study were subjected to an organoleptic assessment on a five-point scale using features, such as clarity (1—not clear, 5—very clear), aroma (1—unpleasant aroma, 5—very pleasant aroma), color (1—unappealing color, 5—very appealing color), taste (1—not tasty, 5—very tasty), and overall impression (1—bad, 5—very good). Participants were also asked to describe the aromas they smelt in beer. Beers were evaluated by a group of 9 trained panelists (21 to 27 years old), which consisted of 6 women and 3 men. Participants were not familiarized with the type of additives used in the study. Samples were given in plastic coded cups with a capacity of 250 mL. The temperature of the served beer was 11 °C.

### 3.6. Data Analysis

Volatile compounds separated from beer were identified by mass spectral analysis, comparing retention indexes (RIs) with Kovats standards (KI exp. and KI lit.) and with NIST11 chemical standard libraries. Two standard matrices were also created (for BC chromatogram and for mango fruit). The chromatograms of the remaining samples (MJ, MP, MR, MRH, and MHH) were integrated using the retention time of the compounds in those two standard matrices, using Mnova MS 12.0.1 software (Mestrelab Research, Santiago de Compostela, Spain). The results of the sensory analysis, polyphenols content, antioxidative activity, basic physico-chemical parameters, and carbohydrate and glycerol content were statistically analyzed in the Statistica 12.5 program from Statsoft (Tulsa, OK, USA) using one-way ANOVA (α = 0.05). Differences between means were calculated using Duncan test (α < 0.05). The results of the volatile compounds analysis are shown as a mean with standard deviation.

## 4. Conclusions

The study indicates that mango fruit can be used as an adjunct, which allows beer with a higher content of volatile compounds and improved aroma to be obtained. The content of volatile compounds was influenced by the type and form in which fruit was added. The beer with mango pulp addition had the greatest volatile component content (2112.14 µg/100 mL) and achieved the best results in the organoleptic analysis in features, such as aroma, taste, color, and overall quality. The addition of mango resulted in beers with a higher polyphenol content and greater antioxidant activity than traditional beer. The mango juice addition had the greatest impact on improving the beer’s antioxidant activity. Homogenisation of mango resulted in beer with higher polyphenol content and improved aroma than the addition of raw mango in pieces, but thermal treatment of the said homogenisate decreased this effect. Furthermore, most beers with mango addition had a lower calorie content than the control sample. Beers with mango addition were also characterized by a lower pH value and extract content, which improves their microbial stability.

## Figures and Tables

**Table 1:** Volatile compounds in mango beers.

			Kovats Indices					BC ^1^	MJ	MP	MR	MRH	MHH
Peak nr	tR (min)	Peak Name	KI exp.	KI Adams	KI NIST	CAS	Chemical Family	µg/100 mL	µg/100 mL	µg/100 mL	µg/100 mL	µg/100 mL	µg/100 mL
1	4.11	Diethyl acetal	717	726		105-57-7	Acetals	2.679 ± 1.088 ^d^	3.0763 ± 1.443 ^a^	3.073 ± 1.274 ^a^	2.822 ± 1.447 ^c^	2.789 ± 1.283 ^c^	2.968 ± 1.055 ^b^
2	4.196	1-Butanol, 3-methyl-	723	736		123-51-3	Alcohols	223.169 ± 42.373 ^a^	177.716 ± 41.276 ^e^	198.286 ± 44.824 ^c^	214.111 ± 38.288 ^b^	190.738 ± 33.288 ^d^	178.409 ± 38.227e
3	4.267	1-Butanol, 2-methyl-	731	739		137-32-6	Alcohols	61.970 ± 28.264 ^a^	55.194 ± 18.934 ^b^	53.748 ± 16.120 ^c^	60.748 ± 25.481 ^a^	56.716 ± 23.943 ^b^	57.472 ± 19.699 ^b^
4	4.768	Propanoic acid, 2-methyl-, ethyl ester	752	756		97-62-1	Esters	1.071 ± 0.688 ^b^	1.214 ± 0.760 ^a^	1.0975 ± 0.799 ^ab^	1.056 ± 0.723 ^b^	1.037 ± 0.606 ^c^	1.114 ± 0.823 ^a^
5	5.138	Isobutyl acetate	769	771		110-19-0	Esters	0.568 ± 0.340 ^c^	0.659 ± 0.439 ^ab^	0.763 ± 0.434 ^a^	0.601 ± 0.307 ^b^	0.728 ± 0.472 ^a^	0.621 ± 0.387 ^b^
6	5.602	2.3-Butanediol	784	788		513-85-9	Alcohols	0.647 ± 0.290 ^c^	0.768 ± 0.399 ^a^	0.659 ± 0.327 ^c^	0.667 ± 0.311 ^c^	0.762 ± 0.381 ^a^	0.710 ± 0.365 ^b^
7	5.841	Butanoic acid, ethyl ester	799	804		105-54-4	Esters	6.793 ± 3.991 ^c^	7.902 ± 4.348 ^b^	8.466 ± 5.007 ^a^	7.640 ± 4.505 ^b^	8.443 ± 4.699 ^a^	7.857 ± 4.875 ^b^
8	6.248	Crotonic acid	835		986(1)	107-93-7	Acids	2.807 ± 1.700 ^c^	3.377 ± 1.791 ^b^	3.627 ± 1.883 ^a^	2.877 ± 1.744 ^c^	3.073 ± 1.800 ^b^	3.041 ± 1.691 ^b^
9	7.135	2-Butenoic acid, ethyl ester	853			10544-63-5	Esters	1.720 ± 0.814 ^c^	1.886 ± 0.986 ^a^	1.917 ± 1.009 ^a^	1.756 ± 0.917 ^c^	1.810 ± 1.106 ^b^	1.809 ± 1.098 ^b^
10	7.92	1-Hexanol	872			111-27-3	Alcohols	0.631 ± 0.368 ^bc^	0.651 ± 0.383 ^b^	0.659 ± 0.401 ^ab^	0.622 ± 0.399 ^c^	0.687 ± 0.309 ^a^	0.666 ± 0.347 ^a^
11	8.117	Isopentyl acetate	872	876		123-92-2	Esters	9.514 ± 4.381 ^b^	9.751 ± 4.889 ^ab^	9.877 ± 5.277 ^a^	9.456 ± 4.268 ^c^	10.182 ± 5.489 ^a^	9.501 ± 4.108 ^bc^
12	8.524	1,3,5,7-cyclooctatetraene	899			629-20-9	Hydrocarbons	0.833 ± 0.484 ^c^	0.868 ± 0.578 ^bc^	0.878 ± 0.583 ^b^	0.830 ± 0.498 ^c^	0.991 ± 0.640 ^a^	0.905 ± 0.614 ^b^
13	8.901	Heptanal	908		901	111-71-7	Aldehydes	0.202 ± 0.092 ^d^	0.255 ± 0.138 ^a^	0.264 ± 0.154 ^a^	0.236 ± 0.141 ^b^	0.227 ± 0.127 ^bc^	0.220 ± 0.147 ^c^
14	9.904	α-Pinene	938	937	939	80-56-8	Monoterpenes	0.660 ± 0.412 ^e^	5.244 ± 2.948 ^b^	5.764 ± 3.088 ^a^	1.284 ± 0.813 ^d^	5.109 ± 2.898 ^b^	4.289 ± 2.669 ^c^
15	10.07	Ethyl β-hydroxybutyrate	941	944		5405-41-4	Esters	0.197 ± 0.119 ^c^	0.241 ± 0.168 ^a^	0.220 ± 0.139 ^b^	0.219 ± 0.142 ^b^	0.224 ± 0.149 ^b^	0.219 ± 0.129 ^b^
16	10.172	Ethyl tiglate	942	939		5837-78-5	Esters	0.635 ± 0.299 ^c^	0.677 ± 0.357 ^b^	0.699 ± 0.409 ^a^	0.632 ± 0.361 ^c^	0.698 ± 0.433 ^a^	0.682 ± 0.381 ^b^
17	10.38	Camphene	950	952		79-92-5	Monoterpenes	0.013 ± 0.009 ^d^	0.031 ± 0.022 ^d^	0.593 ± 0.396 ^a^	0.151 ± 0.087 ^c^	0.551 ± 0.364 ^a^	0.453 ± 0.291 ^b^
18	10.648	Isovaleraldehyde, diethyl acetal	957	955		3842-03-3	Acetals	0.193 ± 0.120 ^c^	0.216 ± 0.145 ^b^	0.240 ± 0.125 ^a^	0.195 ± 0.119 ^c^	0.229 ± 0.131 ^a^	0.221 ± 0.152 ^ab^
19	11.154	1-Heptanol	976	970		111-70-6	Alcohols	0.568 ± 0.331 ^d^	0.659 ± 0.400 ^b^	0.735 ± 0.470 ^a^	0.582 ± 0.348 ^cd^	0.698 ± 0.462 ^ab^	0.600 ± 0.382 ^c^
20	11.251	Acetaldehyde ethyl isoamyl acetal	979	n.d.		13442-90-5	Acetals	0.383 ± 0.255 ^d^	0.512 ± 0.340 ^a^	0.439 ± 0.293 ^c^	0.442 ± 0.300 ^c^	0.426 ± 0.301 ^c^	0.488 ± 0.308 ^b^
21	11.671	5-Hepten-2-one, 6-methyl-	987	987		110-93-0	Ketones	1.219 ± 0.845 ^c^	1.506 ± 0.823 ^a^	1.317 ± 0.923 ^b^	1.186 ± 0.766 ^c^	1.494 ± 1.003 ^a^	1.368 ± 0.881 ^b^
22	11.797	β-Myrcene	994	991		123-35-3	Monoterpenes	51.683 ± 17.111 ^d^	67.152 ± 24.948 ^a^	68.384 ± 22.735 ^a^	63.334 ± 19.641 ^c^	65.269 ± 18.649 ^b^	64.767 ± 25.989 ^bc^
23	12.061	Hexanoic acid, ethyl ester	1001	998	1000	123-66-0	Esters	102.794 ± 26.447 ^d^	109.745 ± 18.748 ^cd^	130.206 ± 32.844 ^a^	105.727 ± 22.900 ^d^	123.241 ± 28.650 ^b^	115.095 ± 26.944 ^c^
24	12.481	3-Hexenoic acid, ethyl ester, (E)-	1009	1007		26553-46-8	Esters	0.202 ± 0.115 ^c^	0.250 ± 0.166 ^b^	0.270 ± 0.147 ^a^	0.204 ± 0.107 ^c^	0.248 ± 0.121 ^b^	0.210 ± 0.099 ^c^
25	12.607	Isobutyric acid, 2-methylbutyl ester	1017	1016		2445-69-4	Esters	0.195 ± 0.085 ^e^	0.257 ± 0.153 ^b^	0.280 ± 0.164 ^a^	0.212 ± 0.0970 ^d^	0.233 ± 0.100 ^c^	0.229 ± 0.109 ^c^
26	12.859	p-Cymene	1027	1025		99-87-6	Aromatic hydrocarbons	0.204 ± 0.121 ^e^	2.313 ± 1.303 ^b^	2.449 ± 1.406 ^a^	0.619 ± 0.382 ^d^	2.371 ± 1.288 ^ab^	1.777 ± 0.990 ^c^
27	13.014	2-Octenal, (E)-	1033			2548-87-0	Aldehydes	8.169 ± 4.667 ^d^	17.727 ± 8.995 ^b^	17.559 ± 9.227 ^b^	15.649 ± 7.606 ^c^	20.664 ± 8.399 ^a^	18.436 ± 7.999 ^ab^
28	13.295	*cis*-β-Ocimene	1043	1038		3338-55-4	Monoterpenes	81.377 ± 16.966 ^c^	128.932 ± 28.307 ^a^	130.943 ± 31.999 ^a^	111.940 ± 19.964 ^b^	112.418 ± 22.838 ^b^	112.026 ± 25.775 ^b^
29	13.631	trans-β-Ocimene	1052	1049		13877-91-3	Monoterpenes	1.436 ± 0.955 ^e^	13.260 ± 6.027 ^a^	14.047 ± 5.934 ^a^	3.397 ± 3.247 ^d^	7.851 ± 3.662 ^b^	6.671 ± 3.100 ^c^
30	13.869	Butyric acid, isopentyl ester	1061	1058		106-27-4	Esters	0.423 ± 0.187 ^d^	0.439 ± 0.200 ^c^	0.494 ± 0.208 ^a^	0.422 ± 0.189 ^d^	0.474 ± 0.213 ^b^	0.444 ± 0.223 ^c^
31	14.062	Ethyl 5-methylhexanoate	1068			10236-10-9	Esters	2.230 ± 0.989 ^d^	2.579 ± 1.299 ^b^	2.741 ± 1.444 ^a^	2.338 ± 1.100 ^cd^	2.467 ± 1.199 ^bc^	2.414 ± 1.201 ^c^
32	14.356	1-Octanol	1075	1071		111-87-5	Alcohols	5.707 ± 3.056 ^bc^	6.036 ± 3.662 ^b^	6.577 ± 4.293 ^a^	5.650 ± 3.601 ^c^	5.335 ± 2.998 ^d^	5.277 ± 2.688 ^d^
33	14.888	Terpinolene	1091	1088		586-62-9	Monoterpenes	0.880 ± 0.623 ^e^	69.098 ± 23.947 ^b^	73.162 ± 22.934 ^a^	16.134 ± 7.993 ^d^	68.373 ± 21.996 ^b^	55.098 ± 18.766 ^c^
34	15.178	4-Heptenoic acid. ethyl ester, (E)-	1099	1090		54340-70-4	Esters	2.161 ± 0.922 ^c^	2.689 ± 1.209 ^a^	2.630 ± 1.189 ^a^	2.414 ± 0.880 ^c^	2.512 ± 1.001 ^b^	2.512 ± 0.996 ^b^
35	15.235	Linalool	1099	1094		126-91-0	Alcohols	2.089 ± 0.998 ^d^	4.609 ± 2.004 ^b^	4.984 ± 2.969 ^a^	4.175 ± 1.866 ^c^	4.731 ± 2.116 ^ab^	4.659 ± 1.889 ^b^
36	15.36	2-Nonen-1-ol	1099	1105		22104-79-6	Alcohols	2.571 ± 1.099 ^d^	2.853 ± 1.283 ^b^	2.982 ± 1.449 ^a^	2.621 ± 1.088 ^c^	2.814 ± 1.204 ^b^	2.828 ± 1.177 ^b^
37	15.642	Phenylethyl Alcohol	1119	1108	1116	22258	Alcohols	218.6835 ± 44.9238 ^a^	184.2365 ± 27.9668 ^d^	209.457 ± 26.844 ^c^	216.614 ± 38.087ab	214.220 ± 29.607 ^b^	209.667 ± 28.931 ^c^
38	16.119	(4E,6Z)-Allo-Ocimene	1127	1131		7216-56-0	Hydrocarbons	1.105 ± 0.662 ^d^	2.386 ± 1.083 ^c^	2.773 ± 1.227 ^a^	2.370 ± 1.198 ^c^	2.414 ± 1.292 ^bc^	2.470 ± 1.302 ^b^
39	16.503	(4E,6E)-Allo-Ocimene	1150	1144		3016-19-1	Hydrocarbons	0.379 ± 0.166 ^d^	0.699 ± 0.3128 ^a^	0.709 ± 0.349 ^a^	0.589 ± 0.261 ^c^	0.659 ± 0.289 ^b^	0.628 ± 0.267 ^bc^
40	16.765	Hexanoic acid, 2-methylpropyl ester	1158	1149		105-79-3	Esters	0.848 ± 0.366 ^d^	0.893 ± 0.501 ^b^	0.940 ± 0.450 ^a^	0.870 ± 0.395 ^c^	0.881 ± 0.466 ^b^	0.878 ± 0.408 ^bc^
41	16.891	2(E)-Nonenol	1163			31502-14-4	Alcohols	0.186 ± 0.0894 ^d^	0.221 ± 0.100 ^b^	0.262 ± 0.100 ^a^	0.200 ± 0.091 ^c^	0.220 ± 0.108 ^b^	0.203 ± 0.088 ^c^
42	17.059	unknown	1171					3.316 ± 1.407 ^e^	3.890 ± 1.682 ^a^	3.906 ± 1.774 ^a^	3.706 ± 1.289 ^d^	3.848 ± 1.277 ^b^	3.731 ± 1.403 ^c^
43	17.224	Camphor	1177	1167		76-22-2	Monoterpenoids	0.304 ± 0.180 ^e^	0.629 ± 0.312 ^d^	0.772 ± 0.380 ^a^	0.652 ± 0.307 ^c^	0.735 ± 0.344 ^b^	0.659 ± 0.321 ^c^
44	17.336	Benzoic acid, ethyl ester	1180	1171		93-98-0	Esters	4.893 ± 2.110 ^e^	6.086 ± 2.553 ^b^	6.398 ± 2.019 ^a^	4.916 ± 2.566 ^e^	5.720 ± 2.990 ^c^	5.487 ± 2.864 ^cd^
45	17.544	Butanedioic acid, diethyl ester	1187	1182		123-25-1	Esters	3.641 ± 1.559 ^d^	4.154 ± 2.245 ^b^	4.302 ± 2.128 ^a^	3.801 ± 1.662 ^cd^	4.050 ± 1.996 ^bc^	3.951 ± 1.849 ^c^
46	17.725	4-Octenoic acid, ethyl ester, (Z)-	1194	1187		34495-71-1	Esters	1.020 ± 0.587 ^d^	1.169 ± 0.749 ^ab^	1.231 ± 0.781 ^a^	1.064 ± 0.681 ^c^	1.132 ± 0.700 ^bc^	1.098 ± 0.664 ^c^
47	17.798	α-terpineol	1196	1190		98-55-5	Monoterpenes	10.315 ± 4.921 ^d^	15.939 ± 6.010 ^b^	16.803 ± 5.449 ^a^	15.080 ± 4.804 ^c^	15.441 ± 5.819 ^bc^	15.209 ± 4.988 ^c^
48	17.91	Octanoic acid, ethyl ester	1203	1197	1196	106-32-1	Esters	709.320 ± 76.334 ^e^	751.453 ± 87.964 ^b^	769.535 ± 94.743 ^a^	723.515 ± 101.885 ^d^	744.916 ± 89.973 ^bc^	737.537 ± 84.962 ^c^
49	18.052	Estragole	1207	1196		140-67-0	Phenylpropanoid	1.520 ± 1.190 ^d^	2.896 ± 1.409 ^b^	3.365 ± 1.663 ^a^	2.607 ± 1.229 ^c^	2.861 ± 1.420 ^b^	2.853 ± 1.366 ^b^
50	18.359	β-Cyclocitral	1231	1220		432-25-7	Monoterpenoids	0.737 ± 0.418 ^e^	1.428 ± 0.800 ^b^	1.493 ± 0.864 ^a^	1.215 ± 0.687 ^d^	1.345 ± 0.763 ^bc^	1.317 ± 0.805 ^c^
51	18.471	Citronellol	1238	1230		106-22-9	Alcohols	1.338 ± 0.742 ^e^	3.752 ± 1.992 ^b^	3.897 ± 1.841 ^a^	3.510 ± 1.316 ^d^	3.686 ± 1.473 ^c^	3.512 ± 1.286 ^d^
52	18.736	Benzeneacetic acid, ethyl ester	1256	1246		101-97-3	Esters	1.521 ± 0.766 ^d^	1.857 ± 1.005 ^ab^	1.937 ± 0.967 ^a^	1.616 ± 0.901 ^c^	1.756 ± 0.969 ^b^	1.646 ± 0.817 ^c^
53	18.779	Isopentyl hexanoate		1252		2198-61-0	Esters	1.269 ± 0.593 ^d^	1.855 ± 0.998 ^ab^	2.054 ± 1.123 ^a^	1.573 ± 0.650 ^c^	1.745 ± 0.811 ^b^	1.756 ± 0.863 ^b^
54	18.918	β-Phenethyl acetate	1271	1258		103-45-7	Esters	19.660 ± 6.163 ^a^	13.486 ± 4.685 ^e^	16.013 ± 4.888 ^c^	17.147 ± 5.944 ^b^	14.724 ± 4.985 ^d^	14.839 ± 5.001 ^d^
55	19.114	1-decanol	1282	1269		112-31-2	Alcohols	1.366 ± 0.628 ^c^	1.536 ± 0.521 ^b^	1.629 ± 0.484 ^a^	1.463 ± 0.489 ^bc^	1.521 ± 0.568 ^b^	1.518 ± 0.471 ^b^
56	19.324	Anethole	1297	1284		104-46-1	Phenylpropanoid	1.333 ± 0.505 ^d^	2.248 ± 1.199 ^b^	2.3670 ± 1.207 ^a^	1.992 ± 0.794 ^c^	2.195 ± 0.975 ^b^	2.004 ± 0.892 ^c^
57	19.747	trans-Geranic acid methyl ester	1339	1324		1189-09-9	Esters	0.788 ± 0.408 ^d^	1.937 ± 0.849 ^b^	2.040 ± 0.661 ^a^	1.547 ± 0.633 ^c^	1.901 ± 0.711 ^b^	1.756 ± 0.686 ^c^
58	19.989	Octanoic acid, 2-methylpropyl ester	1356	1348		5461-06-03	Esters	5.441 ± 2.247 ^d^	6.158 ± 3.162 ^b^	6.897 ± 2.909 ^a^	5.754 ± 3.189 ^cd^	6.146 ± 2.877 ^b^	6.060 ± 2.606 ^b^
59	20.058	Benzenepropanoic acid, ethyl ester		1352		2021-28-5	Esters	0.839 ± 0.427 ^d^	1.098 ± 0.533 ^bc^	1.210 ± 0.642 ^a^	1.050 ± 0.492 ^c^	1.143 ± 0.592 ^b^	1.119 ± 0.467 ^b^
60	20.378	4-Decenoic acid, ethyl ester	1392	1375		76649-16-6	Esters	11.251 ± 4.007 ^d^	13.828 ± 4.993 ^c^	15.648 ± 6.754 ^a^	13.799 ± 5.874 ^c^	14.722 ± 6.0213 ^b^	13.939 ± 5.455 ^bc^
61	20.448	Ethyl decanoate	1399	1395		110-38-3	Esters	127.758 ± 23.633 ^d^	155.833 ± 38.648 ^b^	165.794 ± 44.907 ^a^	143.167 ± 29.976 ^c^	145.303 ± 32.347 ^c^	144.451 ± 28.452 ^c^
62	20.576	Methyleugenol	1408	1402		93-15-2	Phenylpropanoid	1.345 ± 0.885 ^d^	4.714 ± 2.055 ^ab^	4.995 ± 2.129 ^a^	4.073 ± 1.776 ^c^	4.594 ± 1.994 ^b^	4.390 ± 1.859 ^b^
63	20.802	Caryophyllene	1420	1419		87-44-5	Sesquiterpenes	19.057 ± 8.852 ^c^	23.940 ± 9.162 ^a^	24.447 ± 8.995 ^a^	20.736 ± 7.239 ^b^	21.071 ± 7.541 ^b^	20.853 ± 8.014 ^b^
64	20.899	Methyl undecanoate	1420	1426		1731-86-8	Esters	11.780 ± 6.743 ^c^	12.511 ± 5.885 ^b^	14.286 ± 6.551 ^a^	12.342 ± 5.991 ^b^	12.428 ± 5.428 ^b^	12.369 ± 4.894 ^b^
65	20.984	*cis*-Geranylacetone	1435	1435		3796-70-1	Monoterpenoids	2.207 ± 1.249 ^d^	9.455 ± 4.021 ^a^	8.766 ± 4.660 ^ab^	6.581 ± 3.037 ^c^	7.832 ± 3.957 ^b^	7.280 ± 3.616 ^b^
66	21.096	Humulene	1440	1454		6753-98-6	Sesquiterpenes	25.230 ± 7.995 ^c^	30.115 ± 10.078 ^b^	33.441 ± 11.958 ^a^	27.719 ± 8.764 ^bc^	29.851 ± 9.296 ^b^	29.439 ± 9.154 ^b^
67	21.307	Ionone uknown isomer	1455				Monoterpenoids	4.785 ± 2.667 ^d^	15.969 ± 5.496 ^a^	13.964 ± 4.929 ^b^	10.853 ± 4.549 ^c^	11.899 ± 4.687 ^c^	11.042 ± 5.020 ^c^
68	21.975	Undecanoic acid, ethyl ester	1498	1494		627-90-7	Esters	7.561 ± 3.45 ^c^	8.775 ± 5.005 ^ab^	9.580 ± 4.441 ^a^	7.835 ± 3.805 ^bc^	8.126 ± 4.155 ^b^	8.121 ± 3.993 ^b^
69	22.06	Pentadecane	1499	1500		629-62-9	Hydrocarbons	8.450 ± 4.008 ^c^	9.551 ± 5.239 ^ab^	10.210 ± 4.468 ^a^	8.634 ± 3.477 ^bc^	9.264 ± 4.068 ^b^	8.999 ± 3.998 ^b^
		Total concentration						1787.836	1995.034	2112.147	1911.538	2004.959	1946.835

^1^ BC—control beer; MJ—beer with mango juice; MP—beer with mango pulp; MR—beer with raw mango; MRH—beer with raw mango homogenisate; MHH—beer with heated mango homogenisate. Values are expressed as mean (*n* = 2) ± standard deviation. Mean values with different letters (a, b, c, d, e) within the same line are statistically different (*p*-value < 0.05).

**Table 2:** Concentration of carbohydrates and glycerol in mango beers.

Beer Type ^1^	Dextrin Concentration (g/L)	Maltotriose Concentration (g/L)	Maltose Concentration (g/L)	Glucose Concentration (g/L)	Glycerol Concentration (g/L)
BC	34.60 ± 0.89 ^a^	0.24 ± 0.12 ^a^	0.04 ± 0.02 ^a^	n.d.	1.98 ± 0.04 ^c^
MJ	27.72 ± 0.59 ^c^	n.d.	n.d.	n.d.	1.95 ± 0.05 ^c^
MP	28.71 ± 0.65 ^c^	n.d.	n.d.	n.d.	2.31 ± 0.14 ^a^
MR	31.21 ± 0.71 ^b^	0.07 ± 0.07 ^b^	0.03 ± 0.02 ^a^	n.d.	2.09 ± 0.08 ^abc^
MRH	28.92 ± 0.61 ^c^	n.d.	n.d.	n.d.	2.27 ± 0.04 ^a^
MHH	29.18 ± 1.04 ^bc^	n.d.	n.d.	n.d.	2.20 ± 0.05 ^ab^

^1^ BC—control beer; MJ—beer with mango juice; MP—beer with mango pulp; MR—beer with raw mango; MRH—beer with raw mango homogenisate; MHH—beer with heated mango homogenisate. Values are expressed as mean (*n* = 10) ± standard deviation. Mean values with different letters (a, b, c,) within the same column are statistically different (*p*-value < 0.05). N.d.—concentration not detected.

**Table 3:** Basic physico-chemical characteristics of mango beers.

Beer Type ^1^	Wort Extract (*w*/*w*)	Real Extract (*w*/*w*)	Apparent Extract (*w*/*w*)	Alcohol (*v/v*)	Apparent Degree of Fermentation [%]	Real Degree of Fermentation [%]	Calories [kcal]	Density [g/mL]	pH
BC	11.42 ± 0.03 ^b^	3.71 ± 0.03 ^a^	6.74 ± 0.03 ^a^	4.16 ± 0.02 ^d^	41.82 ± 0.21 ^d^	68.40 ± 0.24 ^e^	37.78 ± 0.21 ^ab^	1.015 ± 0.002 ^a^	4.01 ± 0.02 ^a^
MJ	10.81 ± 0.03 ^d^	2.86 ± 0.02 ^e^	5.95 ± 0.02 ^e^	4.13 ± 0.02 ^d^	45.90 ± 0.26 ^a^	74.22 ± 0.18 ^ab^	34.13 ± 0.08 ^d^	1.012 ± 0.002 ^a^	3.77 ± 0.02 ^b^
MP	11.20 ± 0.05 ^c^	3.31 ± 0.02 ^b^	6.41 ± 0.02 ^c^	4.27 ± 0.01 ^c^	43.83 ± 0.17 ^c^	71.30 ± 0.15 ^d^	36.73 ± 0.13 ^c^	1.013 ± 0.001 ^a^	3.67 ± 0.02 ^c^
MR	11.61 ± 0.04 ^a^	3.18 ± 0.02 ^c^	6.49 ± 0.02 ^b^	4.63 ± 0.02 ^a^	45.00 ± 0.26 ^b^	73.21 ± 0.21 ^c^	38.15 ± 0.17 ^a^	1.012 ± 0.002 ^a^	3.60 ± 0.01 ^d^
MRH	11.51 ± 0.03 ^ab^	3.02 ± 0.01 ^d^	6.31 ± 0.02 ^d^	4.62 ± 0.02 ^a^	46.20 ± 0.21 ^a^	74.73 ± 0.26 ^a^	37.44 ± 0.1 ^b^	1.011 ± 0.002 ^a^	3.58 ± 0.02 ^d^
MHH	11.12 ± 0.02 ^c^	2.99 ± 0.03 ^d^	6.32 ± 0.02 ^d^	4.49 ± 0.03 ^b^	44.22 ± 0.25 ^c^	73.81 ± 0.15 ^bc^	36.61 ± 0.27 ^c^	1.011 ± 0.002 ^a^	3.62 ± 0.01 ^d^

^1^ BC—control beer; MJ—beer with mango juice; MP—beer with mango pulp; MR—beer with raw mango; MRH—beer with raw mango homogenisate; MHH—beer with heated mango homogenisate. Values are expressed as mean (*n* = 3) ± standard deviation. Mean values with different letters (a, b, c, d, e) within the same column are statistically different (*p*-value < 0.05).

**Table 4:** Concentration of polyphenols and antioxidative activity in mango beers.

Beer Type ^1^	Polyphenol Concentration	DPPH^•^	FRAP	ABTS^+•^
	mg GAE/L	mmol TE/L	mmol TE/L	mmol TE/L
BC	187.4 ± 6.3 ^a^	1.44 ± 0.10 ^a^	1.04 ± 0.06 ^a^	0.97 ± 0.07 ^a^
MJ	267.6 ± 6.9 ^b^	2.05 ± 0.09 ^b^	1.69 ± 0.14 ^b^	1.74 ± 0.21 ^b^
MP	218.6 ± 4.8 ^c^	1.53 ± 0.07 ^c^	1.32 ± 0.06 ^c^	1.25 ± 0.12 ^c^
MR	233.1 ± 6.1 ^cd^	1.72 ± 0.06 ^d^	1.48 ± 0.07 ^d^	1.27 ± 0.15 ^c^
MRH	243.2 ± 6.8 ^d^	1.78 ± 0.04 ^e^	1.56 ± 0.05 ^e^	1.46 ± 0.08 ^d^
MHH	232.2 ± 2.9 ^cd^	1.72 ± 0.09 ^d^	1.47 ± 0.07 ^d^	1.32 ± 0.13 ^c^

^1^ BC—control beer; MJ—beer with mango juice; MP—beer with mango pulp; MR—beer with raw mango; MRH—beer with raw mango homogenisate; MHH—beer with heated mango homogenisate. Values are expressed as mean (*n* = 12) ± standard deviation. Mean values with different letters (a, b, c, d, e) within the same column are statistically different (*p*-value < 0.05).

**Table 5:** Sensory analysis of mango beers.

Beer Type^1^	Aroma	Color	Clarity	Taste	Overall Impression
BC	2.56 ± 0.29 ^c^	3.00 ± 0.41 ^b^	4.44 ± 0.24 ^a^	3.56 ± 0.24 ^b^	3.44 ± 0.50 ^b^
MJ	4.00 ± 0.33 ^ab^	4.22 ± 0.28 ^a^	3.89 ± 0.20 ^a^	4.00 ± 0.23 ^ab^	4.22 ± 0.22 ^ab^
MP	4.44 ± 0.24 ^a^	4.33 ± 0.33 ^a^	2.78 ± 0.22 ^b^	4.44 ± 0.24 ^a^	4.56 ± 0.18 ^a^
MR	3.44 ± 0.24 ^b^	3.11 ± 0.31 ^b^	4.22 ± 0.24 ^a^	3.78 ± 0.22 ^ab^	3.67 ± 0.24 ^ab^
MRH	4.00 ± 0.23 ^ab^	4.11 ± 0.20 ^a^	3.11 ± 0.33 ^b^	3.89 ± 0.20 ^ab^	3.78 ± 0.32 ^ab^
MHH	3.89 ± 0.42 ^ab^	3.89 ± 0.20 ^ab^	3.00 ± 0.24 ^b^	3.44 ± 0.18 ^b^	3.89 ± 0.39 ^ab^

^1^ BC—control beer; MJ—beer with mango juice; MP—beer with mango pulp; MR—beer with raw mango; MRH—beer with raw mango homogenisate; MHH—beer with heated mango homogenisate. Values are expressed as mean (*n* = 9) ± standard deviation. Mean values with different letters (a, b, c,) within the same column are statistically different (*p*-value < 0.05).

## Supplementary Materials

The following are available online. Figure S1: Mass spectrum of unknown Volatile compounds. Figure S2: Total ion chromatogram of MP.
