# Supplementary material for: Volatile Compounds Content, Physicochemical Parameters, and Antioxidant Activity of Beers with Addition of Mango Fruit (Mangifera Indica)
Source: Molecules. 2020 Jul 2;25(13):3033. doi: 10.3390/molecules25133033 (PMC7411757; doi:10.3390/molecules25133033)

Supplementary Materials

Volatile Compounds Content, Physicochemical Parameters, and Antioxidant Activity of Beers with Addition of Mango Fruit (*Mangifera Indica)*

Figure S1 Mass spectrum of unknown volatile compound (RT 17.059)

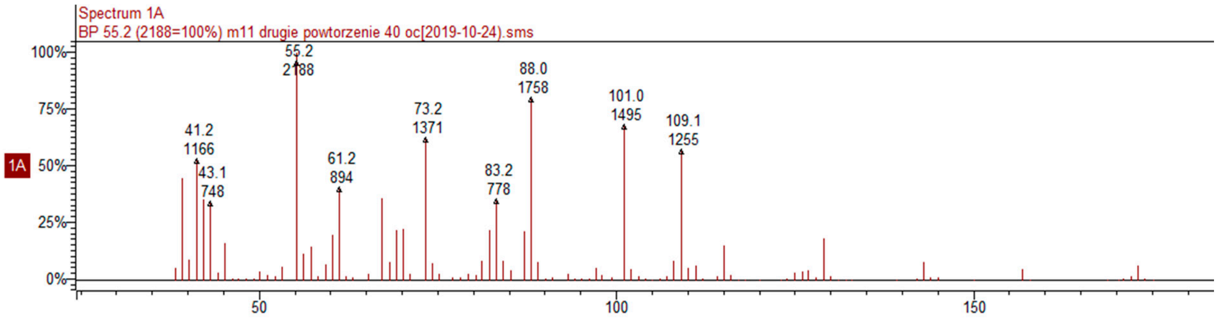

Figure S2 Total ion chromatogram of MP

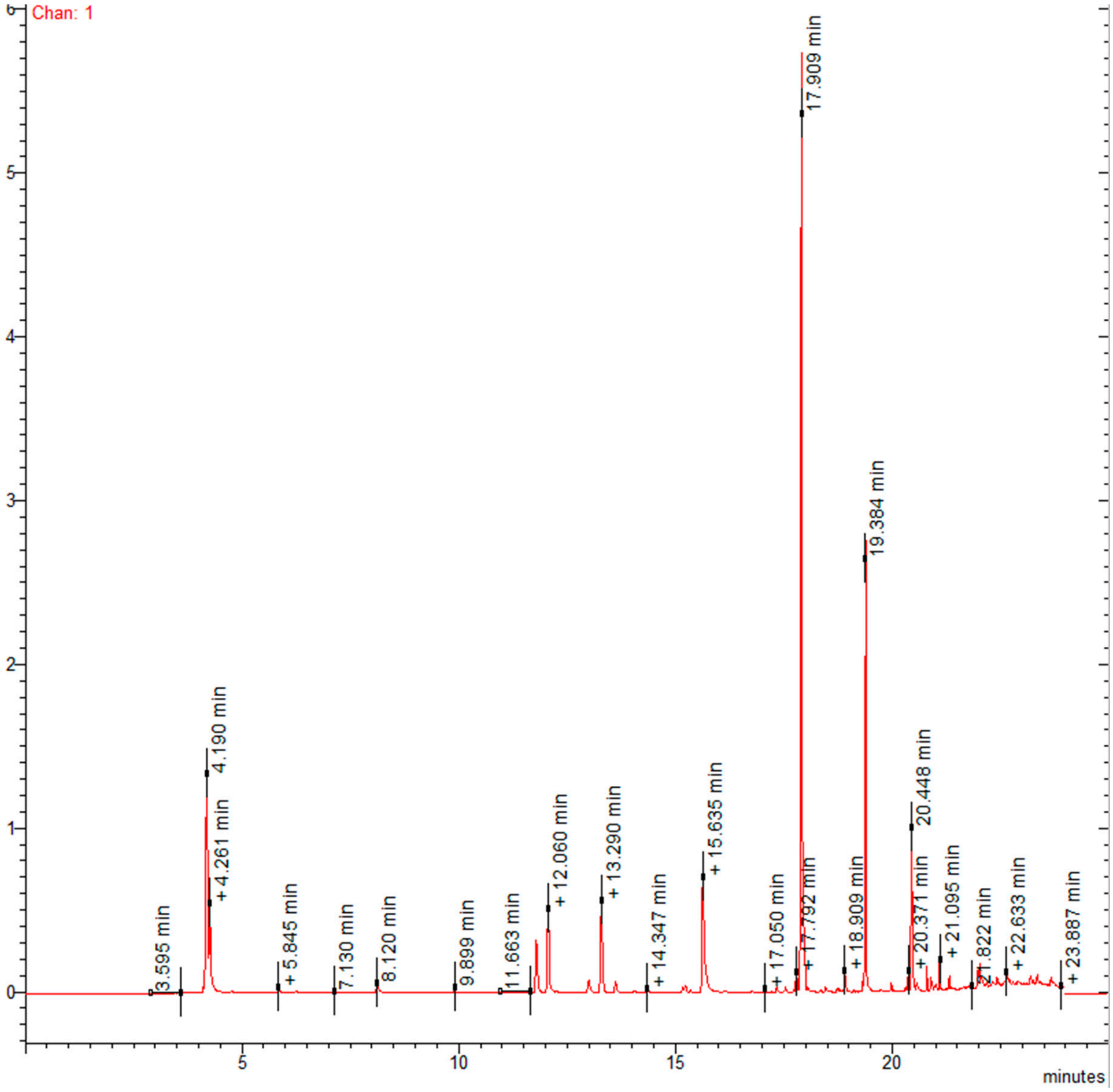

Supplement: Supplementary file 1 [file molecules-25-03033-s001.pdf]
